# When an underestimated zoonosis and antimicrobial resistance collide: Corynebacterium ulcerans

**DOI:** 10.1099/acmi.0.000898.v3

**Published:** 2024-11-22

**Authors:** Giovanni Mori, Giulia Errico, Sara Giancristoforo, Donatella Visentin, Antonella Castagna, Laura Viel, Debora Dellamaria, Giovanni Lorenzin, Gaia Ortalli, Claudio Scarparo, Massimiliano Lanzafame

**Affiliations:** 1 Unit of Infectious Diseases, Ospedale Santa Chiara, APSS, Trento, Italy; 2 Università Vita-Salute San Raffaele, Milano, Italy; 3 Unit of Infectious Diseases, Istituto Superiore di Sanità, Roma, Italy; 4 Hygiene and Public Health Operational Unit, APSS, Trento, Italy; 5 Microbiology and Diagnostic Laboratory, Istituto Zooprofilattico Sperimentale delle Venezie, Villorba di Treviso, Italy; 6 Microbiology and Diagnostic Laboratory, Istituto Zooprofilattico Sperimentale delle Venezie, Trento, Italy; 7 Unit of Microbiology and Virology, Ospedale Santa Chiara, APSS, Trento, Italy; 8 Center for Medical Sciences (CISMed), University of Trento, Trento, Italy

**Keywords:** antimicrobial resistance, *Corynebacterium ulcerans*, diphtheria, public health management, vaccine-preventable diseases, zoonoses

## Abstract

In the European region, diphtheria is now rarely suspected in patients presenting with upper respiratory tract symptoms. *Corynebacterium ulcerans* is the underestimated zoonosis that is replacing *C. diphtheria* infections in industrialized countries, but extensive human and animal prevalence studies are lacking. The range of hosts that can act as reservoirs for *C. ulcerans* is very broad, companion pets currently being the main source of human infection. We report a case of macrolide-resistant * C. ulcerans* infection with no apparent zoonotic transmission and outline the efforts required for the public and zooprophylactic management of these cases. We describe the main critical issues to be addressed to comprehensively tackle this zoonosis in the future.

## Data Summary

All data needed to evaluate the conclusions in this article are included in the paper.

## Introduction

Diphtheria is a potentially life-threatening and vaccine-preventable infection, which is mostly caused by toxigenic *Corynebacterium diphtheriae* strains and occasionally by toxigenic *Corynebacterium ulcerans* and *Corynebacterium pseudotuberculosis* strains [1]. *C. diphtheriae* is re-emerging in multiple world regions: large outbreaks are currently ongoing or have been recently observed in developing countries [2,4] and among migrants in Europe [5]. Despite these somewhat alarming data, in the European region, diphtheria is rarely suspected in patients presenting with upper respiratory tract symptoms. Actually, in vaccinated or partially vaccinated individuals, diphtheria can present without the classic pseudomembrane (membrane-like structures consisting of coagulated exudate that is loosely adherent to an inflamed tissue), and most European laboratories no longer perform routine throat swabs for Corynebacteria. As a result, it is reasonable to assume that diphtheria cases are largely underreported due to a low sensitivity of surveillance systems [1,6].

Moreover, recently, *C. ulcerans* infections have become the most frequent infections due to toxigenic Corynebacteria in industrialized nations where diphtheria is no longer endemic [7,12]. This infection is usually accompanied by mild respiratory and skin manifestations, while more severe manifestations are thought to be related to immunocompromise or inadequate vaccination [1,7, 12]. In the United Kingdom during 1986–2017 and in Japan during 2001–2020, a mortality rate of 6% was reported [7,10]. Human-to-human transmission of *C. ulcerans* has not been established, although a case of dubious inter-human transmission has been reported [13]. In contrast, this species has been isolated from a very broad range of animal hosts and demonstrated a capacity for zoonotic transmission [1,12, 14]. Early reports of *C. ulcerans* human infection were linked to raw milk or dairy product consumption, although recent cases more frequently involve companion animals. Thus, *C. ulcerans* infection is not limited to people involved in animal husbandry and the general population can become infected [12,13]. A recent literature review identified 17 cases of reported *C. ulcerans* zoonotic transmission and compared the few existing national guidelines on the public health management of this infection, highlighting the inconsistency of the latter, especially concerning the management of animal contacts [12].

Antimicrobial-resistant *C. ulcerans* strains have occasionally been isolated from animal and human diphtheria cases [14]. European Committee on Antimicrobial Susceptibility Testing (EUCAST) has recently updated *C. diphtheriae* and *C. ulcerans* evidence-based breakpoints [15], facilitating appropriate antibiotic treatment and prophylaxis, while a global shortage of diphtheria antitoxin continues to be a major problem [1].

Vaccination is the most effective approach to control diphtheria, regardless of the aetiological agent [1]. Whilst almost all European Union/European Economic Area (EU/EEA) countries reported coverage of >90% for the third infant dose of diphtheria toxoid (dTP) vaccines in the last decade [16], the latest seroprevalence studies revealed a lack of vaccine-induced seroprotection against diphtheria in many EU/EEA countries [17,19]. The last Italian seroprevalence study dates back more than 20 years and reported that 40% of the population had inadequate protection (Dt antibodies <0.1 IU ml^−1^) against diphtheria [20]. The largest study conducted in EU/EEA revealed a percentage of sera lacking the protective level ranging from 22.8 to 82.0% [17]. Seroprevalence studies have also unanimously revealed a worsening of titres with increasing age, presumably linked to waning immunity (particularly for diphtheria and pertussis) [17,20]. Such circumstance is probably attributable to the lack of adherence to Centers for Disease Control (CDC) and EU recommendations, also adopted here in Italy with the latest national vaccination prevention plan, which recommends vaccinating adults with a diphtheria toxoid-containing vaccine every 10 years after completing the initial vaccination series in childhood [21].

The dTP vaccines appear to be somewhat active against *C. ulcerans* diphtheria toxin (DT), whereas they do not induce significant levels of antibodies directed against other *C. ulcerans* proteins [22]. The DT produced by *C. ulcerans* and *C. diphtheriae* is similar in function, but there are notable differences in the nucleotide and amino acid sequences, with *C. ulcerans* DT exhibiting ~95% homology to *C. diphtheriae* DT sequences. Variations are mostly in the B fragment of the toxin, impacting cell receptor binding and potentially altering its potency and immune recognition. The *tox* gene encoding DT in both *C. diphtheriae* and *C. ulcerans* is typically located on corynephages, which integrate into conserved chromosomal sites [1,14]. However, *C. ulcerans* also displays a novel pathogenicity island (PAI) capable of carrying the *tox* gene independently of the phage, suggesting a separate pathway for horizontal gene transfer. This mechanism might facilitate a broader spread of the toxin gene among *C. ulcerans* strains, distinguishing it from the strictly phage-dependent toxin acquisition in *C. diphtheriae* [23]. DT divergences between *C. ulcerans* and *C. diphtheriae* could also account for reduced vaccine efficacy [24]. A specific vaccine formulation for *C. ulcerans* and the correlates of immunity with existing dTP vaccines have not yet really been established.

## Case description

In February 2024, a 58-year-old female presenting with sore throat, malaise, cervical lymphadenopathy and pharyngeal pseudomembrane was tested for diphtheria via throat swab (culture on Tryptone Soy Agar with Sheep Blood and Columbia CNA Modified Agar with Sheep Blood), which turned out positive for *C. ulcerans* (identification via *bioMérieux VITEK MS MALDI-TOF* expanded V3.2 database). The patient had not travelled abroad recently; she completed primary vaccination during childhood but then never received booster vaccinations for diphtheria. Antimicrobial susceptibility testing (AST) was performed (Table 1) by broth microdilution, demonstrating macrolide resistance. Although cases of antibiotic resistance have been documented [14], in the dataset of 200 recent clinical isolates of *C. ulcerans* used by EUCAST for the development of the new breakpoints, only one isolate was resistant to erythromycin [15]. The strain was sent to the National Reference Laboratory for Diphtheria at the National Public Health Institute, Istituto Superiore di Sanità (ISS), which confirmed the species identification by the API Coryne test system (*BioMérieux*, Marcy l’Etoile, France) and by real time PCR [25] and the presence of the DT tox gene by real-time PCR. The modified Elek test was performed to confirm the phenotypic expression of the DT. The *C*. *ulcerans* isolate belonged to sequence type (ST) 328. Next-generation sequencing analysis demonstrated that *C. ulcerans* toxin had 95% identity with the toxin of *C*. *diphtheriae* NCTC 13129 by Abricate, using the Virulence Factor Database (VFDB) database. The *C*. *ulcerans* toxin was located in a putative PAI (accession number KP019622), as previously described by Meinel *et al*. [23]. The patient was temporarily quarantined and treated with amoxicillin, obtaining a rapid resolution of the clinical picture. After convalescence, she received a booster with dTP vaccines. Given the presence of mild symptoms, no antitoxin was administered. All six close contacts were tested with nasopharyngeal swabs to exclude *C*. *ulcerans* carriage, then treated with antibiotic prophylaxis (amoxicillin) and vaccinated with dTP vaccines if the vaccination schedule was not up to date. None of them tested positive. The zooprophylactic institute was consulted, and the patient’s three companion cats were tested for *C*. *ulcerans* carriage. Bacteriological examination, matrix-assisted laser desorption ionization-time-of-flight mass spectrometry [26] and 16S rRNA gene sequencing of one nasopharyngeal swab, among three cats tested, were positive for *Corynebacterium spp*. Again, the strain was sent to the ISS for species confirmation and for the detection of the DT tox gene. The isolate was not confirmed as *C*. *ulcerans* by real-time PCR and API Coryne test and did not harbour the DT tox gene. No further analyses were therefore carried out, and the source of infection remains unknown.

**Table 1.** *C. ulcerans* AST: clinical breakpoints according to 2024 EUCAST

Antibiotics	MIC (mg/L)	MIC breakpoints (mg/L)
Clindamycin	3 (R)	0.5
Rifampicin	0.006 (S)	0.06
Erythromycin	0.125 (R)	0.06
Amoxicillin	0.125 (S)	1
Ciprofloxacin	0.047 (S)	0.001–0.5

## Conclusion

The absence of an isolate from the index patient’s companion pets is not unusual in *C*. *ulcerans* cases and therefore does not necessarily rule out the cats as a potential source of the infection [12,14]. It is also possible that transmission to or from one of the six close contacts in this case was missed due to the incubation of the micro-organism in the early stages of infection.

The high proportion of Europeans with unprotected levels of some vaccine-preventable diseases is of concern and has been considered a possible reason for the re-emergence of diphtheria as well. *C*. *ulcerans* infection is one of the many zoonoses to beware of in order not to be unprepared in the coming years. Basic research and further studies are needed to understand the pathogenicity and extent of zoonotic colonization/transmission of this species. AST is of crucial value for initiating appropriate treatment and/or postexposure prophylaxis, considering the occurrence of antibiotic-resistant isolates, particularly in case of resistance to penicillin G and erythromycin, used as the first-line treatment for diphtheria.

Currently, the most important interventions are (1) to increase global surveillance with the urgent institution of national and international guidelines on the public health management of *C*. *ulcerans* to cope with the likely future rise of cases, (2) to maintain laboratory expertise in this specialized area, (3) to promote scientific research to clarify the transmission routes and disease burden of *C*. *ulcerans*, as well as evaluate the possibility of developing a specific vaccine, and (4) to achieve and preserve herd immunity in the migrant, especially ageing population, at high risk of severe disease. To reach this goal, it is necessary to provide information and easy access to primary and particularly booster vaccination.

## Abbreviations

AST: antimicrobial susceptibility testing
EUCAST: European Committee on Antimicrobial Susceptibility Testing
EU/EEA: European Union/European Economic Area
NGS: next-generation sequencing
